# Impact of Spirulina corn soy blend on Iron deficient children aged 6–23 months in Ndhiwa Sub-County Kenya: a randomized controlled trial

**DOI:** 10.1186/s40795-021-00472-w

**Published:** 2021-11-09

**Authors:** Dorothy Apondi Othoo, Sophie Ochola, Elizabeth Kuria, Judith Kimiywe

**Affiliations:** 1 Jomo Kenyatta University of Agriculture and Technology, P.O.Box 62000-00200, Nairobi, Kenya; 2 Kenyatta University, P.O. Box 43844-00100, Nairobi, Kenya

**Keywords:** Iron deficient children 6–23 months. Hematocrit. Randomized controlled trial. Intervention. Spirulina corn soy blend (SCSB)

## Abstract

**Background:**

Iron deficiency anemia (IDA) remains high in Kenya despite interventions. Twenty-seven percent of children aged 6 months-14 years are anemic, with low iron intake (7%) among children aged 6–23 months. Standard food interventions involve a corn soy blend (CSB), which is limited in micronutrients, and fortifiers are not accessible locally. Moreover, the use of spirulina as a strategy for mitigating IDA has not been adequately documented. This study compared the impact of a spirulina corn soy blend (SCSB) on IDA among children aged 6–23 months.

**Methods:**

A total of 240 children with IDA were randomly assigned to study groups at a ratio of 1:1:1 through lotteries, and caregivers and research assistants were blinded to group assignment. Dry-take-home SCSB, CSB and placebo flour (1.7 kg) was given to caregivers to prepare porridges using a flour water ratio of 1:4, producing 600 ml–700 ml of porridge to feed children 200 ml of porridge three times a day for 6 months. Impact was assessed as plasma hematocrit at baseline and after the study. Blood drawing, preparation and analysis were performed in accordance with approved procedures by the EthicsResearchCommittee. Monthly follow-up and data collection on dietary intake, anthropometry, morbidity and infant feeding practices were performed using questionnaires. Relative risk, magnitude of change and log-rank tests were used to compare the impact of the intervention, and significant differences were determined at *P* < 0.05.

**Results:**

The survival probabilities for children consuming SCSB were significantly higher than those consuming CSB (log-rank-X^2^ = 0.978; CI: 0.954–1.033, *P* = 0.001) and the placebo (log-rankX2 = 0.971; CI: 0.943–0.984, *P* = 0.0001). Children consuming SCSB had a mean recovery time of 8 days (CI: 7–12 days) compared to those consuming CSB (19 days; CI: 20–23 days) and placebo (33 days; CI: 3 1–35 days). The recovery rate was 15.4 per 100 persons per day for children who consumed SCSB as opposed to 4.6 and 1.8 per 100 persons per day for those who consumed CSB and the placebo, respectively.

**Conclusion:**

Management of IDA with SCSB compared to CSB and the placebo led to faster reversal and large numbers of recoveries from IDA. The recovery rates were above the World Health Organizations (WHO) minimums standards for food interventions. Efforts to realize high and faster recoveries from IDA should be heightened by fortifying CSB with spirulina powder.

## Background

Iron helps move oxygen from the lungs to the rest of the body and helps muscles store and use oxygen. If a child’s diet lacks iron, he or she might develop a condition called iron deficiency. Iron deficiency in children below 5 years is a common problem globally and more so in the developing countries where diets are composed of unfortified cereals [1]. It can occur at many levels, from a mild deficiency to iron deficiency anemia a condition in which blood doesn’t have enough healthy red blood cells to support sufficient oxygen circulation. Untreated iron deficiency can affect a child’s growth and development and eventual mental retardation [2].

Studies in Kenya indicate low iron consumption (7%) and iron deficiency (26%) among children aged 6–23 months [3]. The disorders can be attributed to a variety of factors including inadequate dietary intakes, low access to adequate and diversified diets, poor infant and young child feeding practices, poor maternal nutrition knowledge and attitudes as influenced by socio-economic and cultural beliefs, poor sanitation and hygiene practices, childhood illnesses and inadequate access to health and nutrition services [4]. These have contributed to increased childhood morbidity, disability, retarded growth and mental development which together derail realization of Kenya vision 2030 and the Sustainable Development Goals. Micronutrient deficiencies acquired during the first 2 years of life are difficult to reverse later in life. The children on complementary feeding are at increased risks of inadequate iron intakes given that their foods are composed of non-haem iron compounds whose bioavailability is greatly influenced by other constituents of the diet consumed [5].

Interventions targeting child nutrition and health remain critical in preventing and reversing IDA including its effects especially among children below 2 years of age. In Kenya, interventions to curb IDA have been conducted using supplemental fortified food products among which Corn Soy Blend (CSB) is the standard food based intervention for iron deficiency while iron and folic acid supplements mostly target pregnant women [6]. The CSB is limited in micronutrients and slow in realizing the expected outcome. Besides, fortifiers used in CSB are not readily available in Kenya making CSB unaffordable for a majority of Kenyans. The processing of CSB is not feasible at household level, a limitation to nutritious food consumption at household level, much as corn and soy bean are locally produced by households. Nutritional impact of supplementary feeding foods on micronutrient deficiencies among children aged 6–23 months in Kenya has not been adequately documented. In addition, information on the potential benefits of spirulina on IDA is scarce. To address these gaps, this study sought to examine whether Spirulina Corn Soy Blend (SCSB) reverses IDA more than the standard CSB [7].

This study aimed to compare impact of SCSB and standard CSB formulations in reversing protein energy malnutrition and iron deficiency among moderately malnourished and iron deficient children within the window of opportunity for correcting malnutrition and micronutrient deficiency disorders. The specific objective was to establish impact of SCSB among iron deficient children aged 6–23 months. It is worth noting that other specific objectives and hypotheses not included in this paper have not been published nor submitted anywhere else for publication.

## Methodology

### Study design and ethics

Study was randomized clinical trial involving interventions with Spirulina-Corn-Soy-Blend (SCSB), Corn-Soy-Blend (CSB) and placebo as consumed by children randomized into 3 arms of parallel study groups. Principal outcome of interest reported in this paper was between arms difference in magnitude of change in hematocrit status and between arms difference in recovery rates from iron deficiency anemia at end of intervention period. The study protocol, data collection procedures and processes were approved by Kenyatta National Hospital/University of Nairobi Ethics-Research Committee (KNH/UON-ERC), original permit number: KNH-ERC/A/240, renewal permit number: KNH/ERC/R/113 and Kenya National Commission for Science, Technology and Innovation (NACOSTI); permit No: NCST/RCD/12A/012/55. Participants signed approved written consent before recruitment into the study.

### Study participants and eligibility criteria

Study participants were protein energy malnourished and iron deficient children aged 6–23 months and their caregivers attending hospitals in the sub-county. Children were screened for eligibility and those who met the following criteria were recruited: WAZ: <−2SD to > − 3SD, MUAC ≥115-125 mm, Hb ≤12 mg/L, pale conjunctiva and > 3 s capillary palm refill, caregiver consent participation, with no pre-existing conditions like cancer, heart, liver and kidney, not in any intervention and caregiver not planning to move out of the study area during study period. Recruitment continued daily for 2 months until sample size was attained. Intervention started immediately following baseline data collection.

### Study settings

The study was done in Ndhiwa Sub-County, Homa-Bay County-Kenya. The Sub-County has eleven level 2 hospitals, two level 3 hospitals and one level 4 hospitals from where screening for eligibility, recruitments and allocation of participants into study groups, blood draw from children participants, nutrition education for caregiver participants, initial cooking demo for porridge and flour ration distributions were done.

### Formulations of Spiralina corn soy blend flour, corn soy blend flour and placebo flour

Both SCSB and CSB flours were based on extruded 80% whole maize, 20% whole soy bean, 0.4% spirulina powder for SCSB flour, 0.4% micronutrient pre-mix for CSB flour, 9% sugar and 0.2% food color. Placebo flour was based on 100% extruded whole maize, 9% sugar and 0.2% food color. All four formulations were designed to meet World Health Organization recommendations for energy for malnourished children Table 1. All flour formulations were identical in color and packaging except packaging sealing strength which was known to Principal Investigator only. Total iron content for SCSB flour was 15.48 mg/100 g dry weight and had its 89.1% HCL extractible. Spirulina powder had total iron content of 15.32 mg/100 g and its 89.6% HCL extractible while CSB and maize flour had total iron contents of 6.15 and 0.81 with extractible HCL of 53.2 and 28.8% respectively. The SCSB flour was highest in iron content. It is worth reporting that SCSB flour was awarded utility model by Kenya Industrial Property Institute (KIPI) utility number; KE/U/2016/612.

**Table 1:** Proximate nutrient contents of spirulina powder, SCSB, CSB and maize flour

Components	Spirulina powder(% ± SD) Kcal/100 g dry weight	% ± SD Kcal/g wet weight	SCSB (% ± SD) Kcal/100 g dry weight	% ± SD Kcal/g wet weight	CSB (% ± SD) Kcal/100 g dry weight	% ± SD Kcal/g wet weight	Maize flour (% ± SD) Kcal/100 g dry weight	% ± SD Kcal/g wet weight
Moisture	2.8 ± 0.1	0	4.4 ± 0.00	0	4.8 ± 0.03	0	5.2 ± 0.00	0
Energy	25.7 ± 31.39	1.1 ± 0.00	54.2 ± 55.25	1.8 ± 0.2	28.8 ± 23.86	1.2 ± 0.1	28.5 ± 39.93	1.0 ± 0.3
Carbohydrate	26.6 ± 0.2	10.6	66.5 ± 0.1	26.6	60.7 ± 0.3	24.0	74.8 ± 0.7	29.9
Protein	49.70 ± 0.1	19.88	61.20 ± 0.1	20.84	38.60 ± 0.3	15.47	1.60 ± 0.1	0.06
Fat	3.03 ± 0.3	0.03	5.20 ± 0.3	0.02	6.16 ± 0.1	0.06	1.47 ± 0.04	0.01
fiber	10.34 ± 0.01	0.04	13.02 ± 0.03	0.02	10.17 ± 0.1	0.04	11.77 ± 0.09	2.01
Iron	15.48 ± 0.3	89.1%*	15.32 ± 0.2	89.6%*	6.15 ± 0.3	52.2%*	0.81 ± 0.2	28.8%*

^a^Values are percentages and means ± standard deviation of triplicate analysis

^b*^ HCL extractable iron

^c^values for iron content was mg/100 g

### Intervention

Intervention was implemented after nutrition education to caregivers on key Infant and Young Child Feeding Practices (IYCF) indicators, identification and nutritional management of PEM and IDA, porridge cooking and feeding index child with porridge using national guidelines for IYCF practices, nutrition education manual for IYCF and Integrated Management of Acute Malnutrition (IMAM) by Ministry of Health (MOH), Kenya [8]. Nutrition Education took place centrally at the Sub-District hospital for 3 days using methods such as lectures, discussions, question answer sessions, demonstrations and role plays. Pre-education test was given to inform of scope of content coverage and post education test to evaluate level of knowledge gained. Key intervention with SCSB, CSB and placebo flours started immediately on conclusion of nutrition education.

#### Experimental group 1

Caregivers of children aged 6–11 months received 1.7 kg of pre-cooked Spirulina Corn Soy Blend flour as distributed by the PI assisted by Nutrition and Nursing Officers. Caregivers were asked to feed their children 200 ml of SCSB porridge 3 times a day for 6 months. This contributed 700Kcal, 175.0 g carbohydrates, 35.1 g protein, 23.6 g fat and 45.9 mg iron per day. Similarly caregivers with children aged 12–23 months received same quantities of SCSB flour and were asked to feed their children 350 ml of SCSB porridge 3 times a day for 6 months. This contributed 1000Kcal, 255 g carbohydrates, 50 g protein, 33 g fat, 70 mg iron per day. The quantities and frequency of feeding were based on WHO/UNICEF Recommended Dietary Allowance (RDA) for energy.

#### Experimental group 2

Caregivers of children aged 6–11 months received 1.7 kg of pre-cooked Corn Soy Blend flour as distributed by the PI assisted by Nutrition and Nursing Officers. Caregivers were asked to feed their children 200 ml of CSB porridge 3 times a day for 6 months. This provided 700Kcal of energy, 52.8 g carbohydrates, 12.3 g proteins, 16.2 g fat and 18.3 mg iron per day. Those with children aged 12–23 months received same quantities of CSB flour and were asked to feed their children 350 ml of CSB porridge 3 times a day for 6 months. This contributed 1000Kcal of energy, 57.6 g carbohydrates, 17.1 g proteins, 26.0 g fat and 25.1 mg iron per day. The children received same quantities of flour and porridge daily as their counterparts in EG1 with different nutrient contents. The quantities and frequency of feeding were based on WHO/UNICEF Recommended Dietary Allowance (RDA) for energy.

#### Control group

Caregivers of children aged 6–11 months received 1.7 kg of pre-cooked placebo flour as distributed by the PI assisted by Nutrition and Nursing Officers. Caregivers were asked to feed their children 200 ml of placebo porridge 3 times a day for 6 months. This provided 700Kcal of energy, 119.6 g carbohydrates, 0.02 g proteins, 0.14 g fat and 0.32 mg iron per day. Those with children aged 12–23 months received same quantities of placebo flour and were asked to feed their children 350 ml of placebo porridge 3 times a day for 6 months. This contributed 1000Kcal of energy, 132.6 g carbohydrates, 1.15 g proteins, 1.25 g fat and 1.82 mg iron per day.

### Data collection and procedures

#### Anthropometry

Measurement of weights and height of children was done monthly on home visits using hanging sprint and length boards, together with age, the measurements were computed into WAZ-score and WHZ-score using ENA for SMART soft ware. The 25 kg hanging sprint graduated to 0.1 kg was used to take children’s weights. Length was measured using height/length board. The readings were recorded in their respective sections of the questionnaire. To obtain children’s weights, the PI assisted by the Nutrition and Nursing Officers had the children weighed in light clothing/pants, the hanging sprint was secured firmly on tree branch then calibrated accordingly. Weighing pant was weighed before placing the child in it and readings recorded to the nearest 0.1 kg. Hanging sprint was calibrated after every measurement and three measurements were done for every child. Length measurements was taken using length board which was placed on a level ground, the child placed lying along the middle of the board on their backs whilst ensuring that heels, knees and head rested firmly on the board and reading recorded to the nearest 0.1 cm.

#### Dietary intakes and nutrient content estimation

Dietary intakes of children was obtained on home using modified 24 h recall and food frequency questionnaire according to the seven food group model by World Health Organization (WHO) guidelines [9] on a monthly basis. Primary caregiver was asked to describe all foods and amounts consumed by children during the previous 24 h and listed accordingly by PI assisted by Nutrition Officers. The PI and Nutrition Officers weighed the actual food samples purchased from local food vendors using SF-400 kitchen scale. Estimation of nutrient intake adequacy was calculated using nutrient content per 100 g of food items and ingredients using Food Composition Table of Kenya by Food and Agriculture Organization (FAO)/Ministry of Health (MOH) [10] and Nutrisurvey2007 software. Energy intakes were compared with the Recommended Nutrient Intake (RNI) by Food and Agriculture Organization (FAO) of the United Nations [11]. Nutrient intakes of breast fed children were calculated separately according to average breast milk intake; equivalent to 533 ml per day as documented by Dowey and Brown [12]. Nutrient Adequacy Ratio (NA R) of a given nutrient was estimated as ratio of actual nutrient intake per day for sex and age cohorts. Nutrient intakes were classified as inadequate (<66%), fair (66% to <100%) and adequate intakes (≥100%). Mean adequacy ratio was calculated as sum of all NARs at 100% [13]. Median of nutrient adequacy ratio was calculated as proxy composite indicator for micronutrient adequacy and compared with RNI for age and sex cohorts according to FAO [13]. Dietary Diversity Scores (DDS) was estimated for individual child by summing all food groups consumed based on seven food groups (starchy staples, legumes, meat, dairy products, nuts, eggs, Vitamin A rich fruits and vegetables, other fruits and vegetables and iron rich or iron fortified foods) according to guidelines from WHO/FAO [14]. The DDS were classified as low DDS (<4 food groups), moderate DDS (4 to 7) and High DDS (≥7). The MMF was based on reported number of meals while MAD on proportion of breast fed children who had at least M DD and MMF the previous 24 h and non-breast fed children who received at least 2 milk feeding, at least MDD and MMF the previous 24 h according to WHO indicators for assessing IYCF practices [15].

#### Blood draw and analysis

Blood samples for measurements of iron (Hematocrit) and protein energy (Retinol Binding Protein) status of children were drawn from by trained medical laboratory technicians of various hospital laboratories at baseline and end line. Technicians drew blood through single venipuncture using gauge 23 single use needles with a small volume and low vacuum evacuated tube, care was ensured to obtain free flow of blood into a clearly labeled tube and no more than 5 ml of blood was drawn from each child as recommended by World Health Organization [16]. Collected blood samples was labeled with the codes assigned for each child’s identification and stored in snugly filled in stackable trays in coolants at 4 °C and centrifuged at a temperature of 15 °C and spun at 2000 revolution per minute for 10 min within 45–60 min of blood draw. Separation of plasma from the blood cell was done and transferred into storage tubes which were labeled with child’s ID and immediately stored in liquid nitrogen at -70 °C awaiting analysis at Kenya Medical Research Institute (KEMRI)/Walter reed laboratory-Kericho. The blood draw and sample preparation at the end of study followed the same procedure. Hematocrit (Hct) levels were analyzed using immunoassay method. Levels of ≤20% was considered as severe iron deficiency, 21–35% as moderate iron deficiency while 36–39% as mild and 40–45% as normal levels free from IDA. Analysis involved a total blood count to determine hemoglobin from which proportion of hematocrit is calculated. The frozen specimen was thawed to 37 °C and drawn by the syringe into the cuvette within the gas analyzer maintained at 37 °C. Thereafter, 1 μL of the specimen was hemolyzed via ultrasound (30 kHz). Hemoglobin content was assessed spectrophotometrically using different wave lengths (478 to 672 nm). The light was transmitted via glass-fiber optics through a diffraction gating, which diffracts the light into 128 single wavelengths. Hemoglobin content (ctHB) of the blood sample was determined using Lambert-Beer equation. Hematocrit (Hct) was calculated based on the ctHb using an internal algorithm.

#### Maternal IYCF practices

Observation on child feeding and other maternal child care practices were done by the PI assisted by Nutrition and Nursing Officers randomly using observation check list on a monthly basis, the observations lasted for 6 hours in any single day and focused on child’s expressed feeding cues on intervention porridge, time gap between meals, food served and portions and other Water Sanitation and Hygiene (WASH). Maternal perceptions on the recommended IYCF practices were also captured in the Focus Group Discussion (FGD) guide.

#### Morbidity

Morbidity of children was assessed using semi-structured questionnaires with both open and close end questions by the PI assisted by Nutrition and Nursing Officers monthly on home visits. The questions focused on symptoms of sicknesses 2 weeks prior interviews, frequency and actions taken during sickness.

#### Acceptability of intervention

Acceptability of intervention was assessed based on maternal perceptions, practice and sensory evaluation of the porridges using FGDs guide and sensory evaluation questionnaire at end of intervention. Attributes assessed included color, aroma, taste, consistency and mouth feel. Options for evaluation ranged from excellent, very good, good, worse and worst. Perceptions and practice characteristics such as willingness to feed children on flours after the study, willingness to recommend use of flours to others and ability to identify physical benefits of flours. Children’s degree of liking porridge included attributes such as highly liked, liked, tolerated and not liked besides observed cues during feeding as reported by the caregivers. Three FGD were conducted per study group at baseline and two FGDs; one for caregivers of children in SCSB and the other for those in CSB at the end of study. The FGDs consisted of 12 mothers randomly sampled from each study group using lottery and moderated by PI, Nutrition and Nursing officers.

#### Follow up

Principal Investigator (PI) together with Nutrition and Nursing Officers did random observations on caregivers uptake of intervention besides monthly data collection and assessment of the following indicators: child consumption of study porridge, weight, height, age, WHZ-score, WAZ-score, introduction of solid, semi-solid or soft foods, Minimum Meal Frequency (MM F), Minimum Dietary Diversity (MDD), Minimum Acceptable Diet (MAD), consumption of iron rich or iron fortified foods, observed child’s cue while feeding on study porridge, occurrence and frequencies of child illness especially diarrhea, fevers and Acute Respiratory Illness (ARI). Information on acceptability of intervention and blood sample for analyzing Hematocrit (Hct) status and Retinol Binding Protein (RBP) was collected at the end of 6 months of intervention.

### Sample size determination

With 80% statistical power at 5% level of confidence and effect size of 0.8 predicted changes in Protein Energy Malnutrition (PEM), we included 84 participants in each arm of the study based on the formula by Kesley et al. [17]. Sample size was subjected to correction factor of 10% to cater for attrition using formula by Fleiss [18] each arm of the study had 92 participants making a total sample size of 276 participants.

#### Randomization

**Fig. 1:**
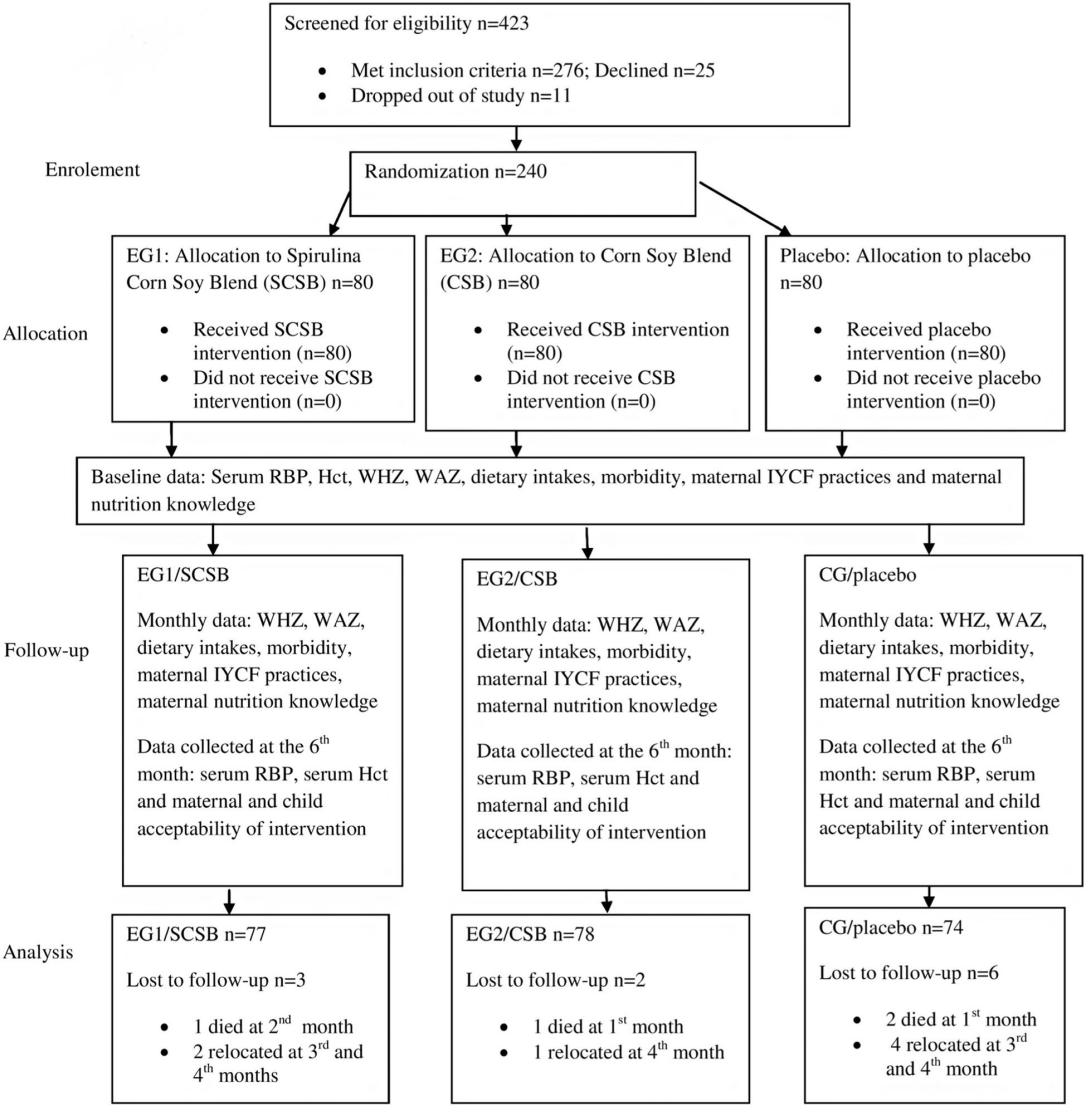
Randimization and flow of the participants throughout the study

Randomization of participants was done using lottery. Random numbers in the calculated sample size (1–276) was computer generated, printed, cut into same size, folded in same pattern and mixed in an opaque containers with a lid and care givers asked to pick one at a time. Each participant handed over the piece of paper to the Principal Investigator (PI) who unfolded the pieces and registered to the coinciding groups in 1:1:1 ratio. Total of 36 caregivers declined to participate and remaining 240 were randomly assigned groups. Those who picked numbers 1–80 belonged to Experimental Group 1 (EG1) and their children received SCSB while those who picked numbers 81–160 and 161–240 belonged to EG2 and Control Group (CG) receiving CSB and placebo respectively as shown in Fig.1. Blinding was done to caregivers, Nutrition Officer, Nursing officer and Laboratory Technician by the Principal Investigator who was the only one with knowledge of group assignments and treatments. Intervention flours were similar in color and packaging.

### Data analysis

Quantitative data was analyzed using Statistical Package for Social Sciences (SPSS) version 20. Descriptive statistics such as frequencies, means, medians and standard deviations were used to describe demographic and socio-economic characteristics of the children and the caregivers. Inferential statistics was used to determine similarity in the baseline characteristics of study groups, relationships and associations between variables. Analysis of Variance (ANOVA) test was used for relationships between categorical variables such as morbidity, caregiver’s nutrition knowledge, IYCF and WASH practices, nutrition knowledge and acceptability of intervention. Relative Risk (RR), Magnitude of change and log-rank-test was used to test for the impact of SCSB on PEM and IDA of the children and significant differences between the intervention groups and the control group was determined at a significance of *P* < 0.05. Qualitative data was summarized into themes, triangulated and contextual analysis done.

## Results

The children who completed the study and their data analyzed were 229 children; thus, 77, 78 and 74 children in SCSB, CSB and placebo respectively as shown in Table 2.

**Table 2:** Monthly attrition rates

Month	EG1 n (%)	EG2 n (%)	CG n (%)	Total
Baseline	80 (100)	80 (100)	80 (100)	240 (100)
Month 1	80 (100)	79 (98.8)	78 (97.5)	237 (98.8)
Month 2	79 (98.8)	79 (98.8)	78 (97.5)	236 (98.3)
Month 3	78 (97.5)	79 (98.8)	76 (95.0)	233 (97.1)
Month 4	77 (96.3)	78 (97.5)	74 (92.5)	229 (95.4)
Month 5	77 (96.3)	78 (97.5)	74 (92.5)	229 (95.4)
Month 6	77 (96.3)	78 (97.5)	74 (92.5)	229 (95.4)

### Characteristics of children and caregivers at baseline

The mean age for children were 14.7(5.1) and 45.4% were males while 20.8% were first born. The mean age of caregivers was 27.5(9.1), 11.7% were males while 87.1 and 91.7% were married and biological parents respectively. About 76.8%, *n* = 42 of caregivers had no formal education and 56.25%, *n* = 1 35 were self employed. Mean monthly income for the caregivers was 1724.3 5(0.7). Randomization was successful as reflected in the equivalence in the demographic and socio-economic characteristics assessed at baseline and more so no significant differences between study groups at base line, Table 3.

**Table 3:** Characteristics of study participants

Characteristics	SCSB *N* = 80	CSB *N* = 80	Placebo *N* = 80
Age of children month (Mean SD)	14.1 (5.0)	14.9 (5.2)	14.6 (4.9)
Sex: n (%) Male	43 (53.7)	34 (42.5)	33 (41.3)
Female	37 (46.3)	46 (57.5)	47 (58.7)
Birth order n(%) First born	15 (18.7)	17 (21.3)	18 (22.5)
Other	65 (81.3)	63 (78.7)	62 (77.5)
WHZ: Mean (SD)	−2.1 (0.5)	−2.3 (0.4)	−2.2 (0.5)
WAZ: Mean (SD)	−2.2 (0.3)	− 2.3 (0.6)	− 2.3 (0.3)
MUAC: Mean (SD)	122.9 (0.3)	122.6 (0.2)	122.8 (0.2)
RBP status: mean (SD)	0.046 (0.1)	0.051 (0.1)	0.050 (0.1)
Breast feeding status: n(%) BF	49 (39.2)	55 (44.0)	50 (20.0)
Not BF	3 1 (24.8)	25 (20.0)	30 (24.0
Age of caregiver: Mean (SD)	28.7 (8.6)	27.7 (9.1)	29.9 (11.3)
Sex: n(%) male	5 (6.2)	11 (13.7)	12 (15.0)
Female	75 (93.8)	69 (86.3)	68 (85.0)
Marital status: n(%) Married	71 (89.9)	70 (87.5)	68 (85.0)
Others	9 (11.1)	10 (12.5)	12 (15.0)
House hold size: mean (SD)	12 (2.6)	10 (3.8)	12 (2.9)
Number of U5s: Mean (SD)	3 (1.0)	4 (1.9)	3 (1.3)
Relationship to child: n(%) Own child	76 (95.0)	7 1 (89.7)	73 (9 1.3)
Other	4 (5.0)	9 (11.3)	7 (8.8)
Education level n(%) No education	15 (19.0)	8 (10.5)	19 (22.4)
Primary	42 (53.2)	46 (58.1)	40 (52.9)
Secondary	18 (22.8)	20 (26.3)	17 (20.0)
Mid level college	4 (3.8)	4 (3.8)	3 (3.5)
Graduate	1 (1.2)	2 (1.3)	1 (1.2)
Occupation n(%) Waged labor	2 (2.5)	3 (3.9)	2 (2.4)
Self employed	44 (55.6)	46 (20.2)	45 (57.8)
Not employed	34 (41.9)	3 1 (40.8)	33 (39.8)

^a^Age for children expressed in months

^b^Age for care givers expressed in years

### Impact of SCSB on iron deficient children aged 6–23 months

**Fig. 2:**
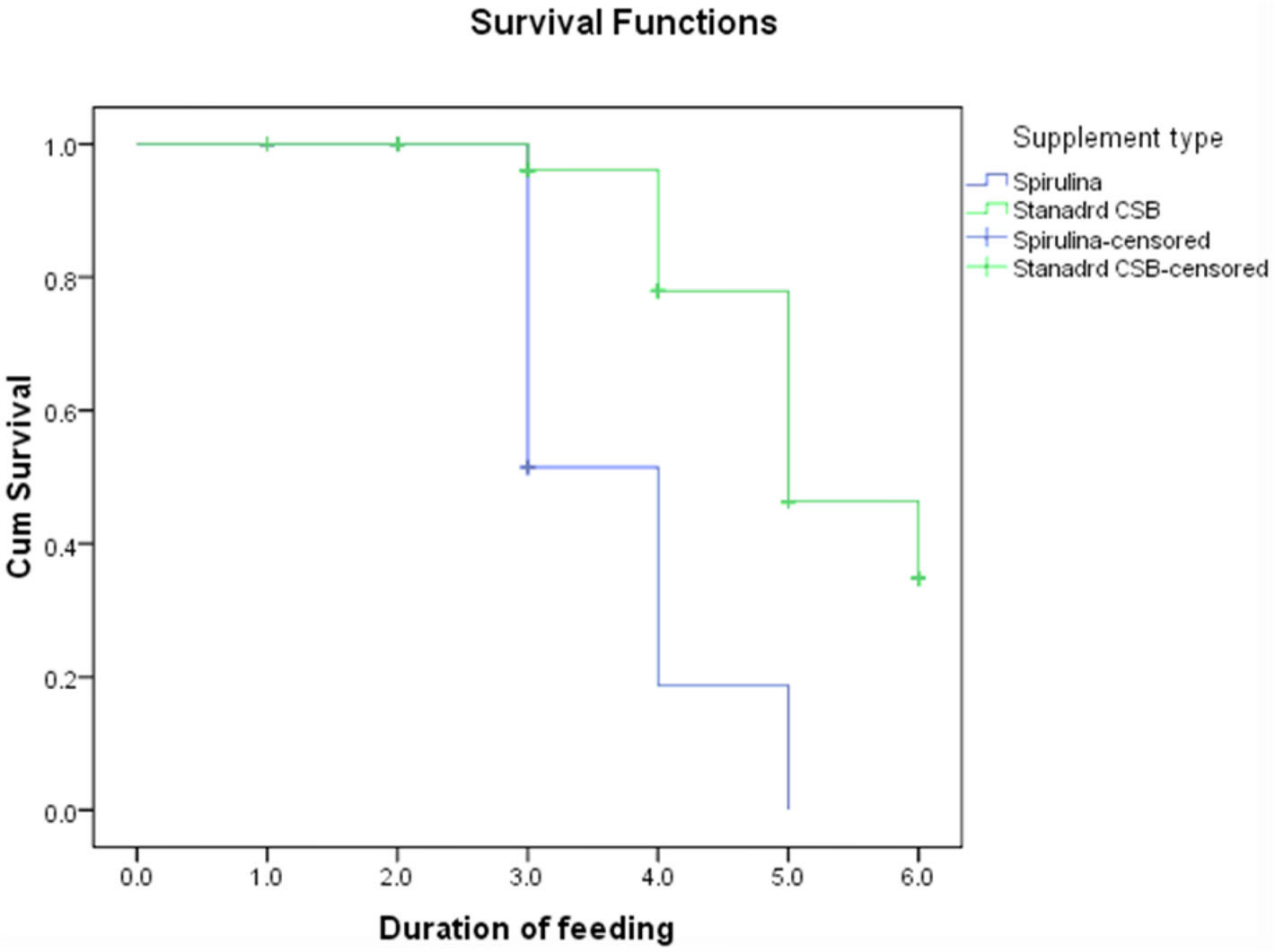
Recovery rates of the children

The children who consumed SCSB had the highest mean magnitude of change (4.126 ± 0.313) in Hct, followed by children who consumed CSB (1.241 ± 0140) while magnitude of change for those who consumed placebo was 0.48 1 as indicated in Table 4. Children who consumed SCSB significantly improved in Hct compared to those who consumed CSB (Difference-in-Difference; DID 3.775; CI 0.41–0.84, *P* = 0.001) and placebo (Difference-in-Difference; DID 4.835; CI 0.27–0.71; *P* = 0.000). However, there were no significant difference between Hct status of children who consumed CSB and placebo (DID 0.106; CI 0.361–0.172; *P* = 0.088) as indicated in Table 4. Children who consumed SCSB were 3.1 and 4.0 times likely to recover from iron deficiency earlier compared to those who consumed CSB (Relative Risk; RR =3.15; CI: 1.91–2.07, *P* = 0.002) and placebo (RR = 4.07; CI: 3.66–3.79, *P* = 0.0001) as indicated in Table 4. Survival probabilities for children consuming SCSB were significantly higher than the survival probabilities of those who consumed CSB (Log-rank-X^2^ = 0.978; CI: 0.954–1.033, *P* = 0.001) and placebo (Log-rank-X^2^ = 0.971; CI: 0.943–0.984, *P* = 0.0001), suggesting survival benefit at the third month as indicated in Table 4 and Fig. 2. Children who consumed SCSB had mean recovery time of 8 days; CI: 7–12 compared to children who consumed CSB (19 days; CI: 20–23) and placebo (33 days CI 31–35) as shown in Table 4. Recovery rate was 15.4 per 100 persons per day for children who consumed SCSB as opposed to 4.6 and 1.8 per 100 persons per day for those who consumed CSB and placebo respectively as shown in Table 5 and Fig. 2.

**Table 4:** Magnitude of change in Hct status of the children

Indicators Hct Study group	Mean Baseline mean	End line mean ANOVA(1 SE)	Magnitude of change	*P*-value
SCSB	0.646 ± 0.1	4.126 ± 0.31	19.067 (0.2)	0.001*
CSB	0.691 ± 0.1	1.241 ± 0.14	8.12 (0.4)	0.410
Placebo	0.650 ± 0.1	0.181 ± 0.33	-12.9 (0.4)	0.656
	Baseline	End line	DID (95% CI)	*P*-value
	difference	Difference		
Mean of SCSB minus mean of CSB	−0.005	2.885	3.775	0.001*
Mean of SCSB minus mean of Placebo	−0.004	3.945	4.835	0.0001*
Mean of CSB minus mean of placebo	0.001	1.06	0.106	0.088
		RR(95%CI)	Z statistics	*P*-value
SCSB n = 4 Vs CSB n = 55		3.15 (1.91–2.07)	8.12	0.002*
SCSB n = 4 Vs placebo n = 64		4.07 (3.63–3.76)	12.45	0.0001*
CSB n = 55 Vs placebo n = 64		1.77 (1.68–1.76)	4.61	0.182
Placebo n = 64 (86.5%)		1		
		X^2^-Log-rank (95%CI)		*P*-value
SCSB Vs CSB		0.978 (0.954–1.033)		0.001*
SCSB Vs placebo		0.971 (0.943–0.984)		0.0001*
CSB Vs Placebo		1.901 (0.881–0.933)		0.701
Placebo		1		
		Mean recovery time in days (CI)		*P*-value
SCSBVs CSB		8 (7–12)		0.002*
SCSB Vs placebo		19 (20–23)		0.001*
CSB Vs Placebo		33 (31–35)		0.002*
Placebo		1		

^a *1*^*SE*: Type 1 Standard Error

^b *^: Significant at *P* < 0.05

**Table 5:** Survival rate of children over study period

Study group	Time interval in months	Those at risk during interval N_t_	Average Number at risk of IDA during interval, N_t*_	Number Recovering from IDA during interval, R_t_	Lost to Follow-up, C_t_	Proportion Recovering from IDA during interval, q_t_	Among those at risk, proportion surviving during Interval, p_t_	Survival Probability S_t_
SCSB	0	80	40					
	1	73	73	7		5.39	4.39	4.39
	2	56	36	16	1	12.32	11.32	49.70
	3	32	15.5	23	1	17.71	16.71	81.51
	4	9	4	22	1	16.94	15.94	94.42
	5		4.5	9		6.93	5.93	100.00
	6					0.00	0.00	100.00
CSB	0	80	40					
	1	79	39		1			
	2	78	39	1		0.78	0.22	0.22
	3	77	38.5	1		0.77	0.23	2.05
	4	73	36	3	1	2.19	1.19	3.61
	5	69	34.5	5		3.45	2.45	4.76
	6	59	29.5	10		5.90	4.90	5.49
Placebo	0	80	40					
	1	79	39		1	0.00	0.00	0.00
	2	78	38.5		1	0.00	0.00	0.00
	3	77	38		1	0.00	0.00	0.00
	4	75	37	1	1	0.75	0.75	0.75
	5	74	37		1	0.00	0.00	0.75
	6	72	36	1	1	0.72	0.72	0.54

Table uses the actuarial method to construct the follow-up life table where the time is divided into equal intervals

## Discussion

In this study, interventions with Spirulina Corn Soy Blend (SCSB) yielded higher and faster recoveries from iron deficiency among children more than Corn Soy Blend (CSB) and placebo. This can be attributed to the high iron contents of spirulina powder used to fortify CSB and high bioavailability though not analyzed in this study. Previous related studies have reported positive association of food interventions with improved nutritional status. A study by Ware that analyzed digestibility of spirulina powder showed that consumption of 10 g of spirulina powder per day yield 55% iron that is 60 times readily absorbed compared to iron supplements [19]. A study by Mike that administered 1 0 g of spirulina powder to adolescent girls indicated improved hemoglobin levels compared to those who took iron supplements [20].

Another study by Kager that administered 3 g spirulina powder to 3 year old children living with HIV in Bukina Faso reported significant reduction in anemia among the children [21]. Dikosso and Onana in their study reported improved High Density Lipoprotein levels among HIV infected adults with a heart condition fed on 10 g spirulina per day for 24 consecutive weeks in Sub-Saharan Africa [22]. Ziaciddin found that daily supplementation with iron sprinkles significantly managed iron deficiency more than the weekly supplementation with iron ferrous tablet among iron deficient children in Bangladesh [23]. A study by Mangitsu on dietary diversity and anemia among children 6–23 months in Welaita Ethiopia showed that children with high dietary diversity scores had lowest risks of anemia [24]. A study by Slotzfus on effects of spirulina on anemia among patients with poor spleen functions realized significant reduction in anemia among patients who consumed 10 g spirulina powder compared to those who consumed lean meat [25].

Despite of reported micronutrient contents of spirulina, available evidence from placebo- controlled randomized trial among patients with immune disorders showed that the episode of recoveries were not significantly different between *spirulina* and placebo [26]. Information on the intervention and nutrition contents of spirulina and placebo in this study was not provided as well as indicators for analysis. A study by DeArruda that administered Spirulina at a dose of 5 g a day did not find significant difference in any of the four subjects and possibly spirulina had no effect on opportunistic infections. This could be due to the dosage level and logistics for adherence to intervention given that no information is provided on the recommended dosage of spirulina for adults and details of intervention [27]. Azabji’s study compared albumin status of HIV infected patients consuming spirulina and soy based foods and reported slight increase in albumin levels in both groups however the levels remained constant over the 12 weeks intervention with no significant differences in the albumin levels. This could be due to the fact that the study sample was HIV positive individuals on various Anti Retrovial Therapies that could affect albumin status differently. Their protocol did not provide information on analysis of parameters with respect to antiretroviral therapies [28]. Reddy et al. on the effect of spirulina on allergic rhinitis showed that high dose of *Spirulina* significantly reduced IL-4 levels, zinc status and underweight [29]. Our study compares closely with Mani et al. that reported significant effect of spirulina based cereal blend on iron deficiency anemia among patients on chemotherapy [30]. Similarly Golden et al. showed improvement in hemoglobin levels among patients supplemented with spirulina for 12 weeks [31]. Yameni et al. however did not realize significant difference in nutrition status among patients supplemented with spirulina for 3 months. This could be due to the fact that Yameni’ s study did not consider nutritional intervention among patients and also the indicator used for assessing nutrition status was not appropriate for monitoring progress [32].

Our study showed significantly higher and faster recovery rates from I DA with consumption of Spirulina Corn Soy Blend which compares with Kulshrethsha who showed improvement in iron status among patients on spirulina compared to those who consumed micronutrient premix soy blend [33]. Amha also reported improvements on nutritional status among patients on spirulina supplements though the patients were not on food interventions as the case in our study [34]. Simpore et al. reported improvements on nutrition status of pregnant women consuming spirulina powder and soy beans blend noting that soy bean based foods also improves nutrition status though slowly [35]. Lazzerini et al. reported increased likelihood of recovery from acute malnutrition among children consuming specially formulated complementary foods as opposed to those on nutrition education and counseling [36]. Lin’s study did not show recoveries from anemia among malaria survivors on spirulina tablet and soy based blends. The study did not indicate consumption quantities for the soy blend nor the intervention procedures [37].

## Conclusions

With these findings, we have showed that spirulina can be used to fortify CSB to improve its micronutrient contents and that treatment of IDA with SCSB is possible at household level and boosts iron status of children than CSB. The information provided is valuable in strategizing for food based programs targeting child malnutrition and contribute towards efforts in addressing micronutrient deficiencies through fortification of staples using locally produced fortificant and possible fortification at house hold level.

### Trial registration

Registration of this RCT was done retrospectively at Pan Africa Clinical Trial Registry on 28 Apr 2020, registration number: PACTR202004842786087.

## Data Availability

All the data set used and/or analyzed during the current study are included in this manuscript, Pan Africa Clinical Trial Registry and in an attached supplementary data file.

## Abbreviations

SCSB: Spirulina Corn Soy Blend
IDA: Iron Deficiency Anemia
CSB: Corn Soy Blend
KNH-UoN ERC: Kenyatta National Hospital-University of Nairobi Ethical Research Committee
NACOSTI: National Commission for Science Technology and Innovation
WAZ: Weight-for-Age Z-score
SD: Standard Deviation
MUAC: Mid Upper Arm Circumference
Hb: Hemoglobin
PEM: Protein Energy Malnutrition
PI: Principle Investigator
EG: Experimental Group
CG: Control Group
IYCF: Infant and Young Child Feeding
IMAM: Integrated Management of Acute Malnutrition
MOH: Ministry Of Health
WHZ: Weight-for-Height Z-score
MMF: Minimum Meal Frequency
MDD: Minimum Dietary Diversity
MAD: Minimum Acceptable Diet
ARI: Acute Respiratory Infections
Hct: Hematocrit
RBP: Retinol Binding Protein
WHO: World Health Organization
RNI: Recommended Nutrient Intake
FAO: Food and Agriculture Organization
NAR: Nutrient Adequacy Ration
DSS: Dietary Diversity Score ct
HB: Hemoglobin Content
WASH: Water Sanitation and Hygiene
FGD: Focus Group Discussion
SPSS: Statistical Package for Social Sciences
ANOVA: Analysis of Variance
DID: Difference-in-Difference
RR: Relative Risk
CI: Confidence interval
HIV: Human Immunodeficiency Virus
